# Canola Oil in Lactating Dairy Cow Diets Reduces Milk Saturated Fatty Acids and Improves Its Omega-3 and Oleic Fatty Acid Content

**DOI:** 10.1371/journal.pone.0151876

**Published:** 2016-03-25

**Authors:** Katiéli Caroline Welter, Cristian Marlon de Magalhães Rodrigues Martins, André Soligo Vizeu de Palma, Mellory Martinson Martins, Bárbara Roqueto dos Reis, Bárbara Laís Unglaube Schmidt, Arlindo Saran Netto

**Affiliations:** 1 Department of Animal Science, School of Animal Science and Food Engineering, University of São Paulo, Pirassununga, São Paulo, Brazil; 2 Department of Nutrition and Animal Production, School of Veterinary Medicine and Animal Science, University of São Paulo, Pirassununga, São Paulo, Brazil; Medical University of South Carolina, UNITED STATES

## Abstract

To produce milk that is healthier for human consumption, the present study evaluated the effect of including canola oil in the diet of dairy cows on milk production and composition as well as the nutritional quality of this milk fat. Eighteen Holstein cows with an average daily milk yield of 22 (± 4) kg/d in the middle stage of lactation were used. The cows were distributed in 6 contemporary 3x3 Latin squares consisting of 3 periods and 3 treatments: control diet (without oil), 3% inclusion of canola oil in the diet and 6% inclusion of canola oil in the diet (dry matter basis). The inclusion of 6% canola oil in the diet of lactating cows linearly reduced the milk yield by 2.51 kg/d, short-chain fatty acids (FA) by 41.42%, medium chain FA by 27.32%, saturated FA by 20.24%, saturated/unsaturated FA ratio by 39.20%, omega-6/omega-3 ratio by 39.45%, and atherogenicity index by 48.36% compared with the control treatment. Moreover, with the 6% inclusion of canola oil in the diet of cows, there was an increase in the concentration of long chain FA by 45.91%, unsaturated FA by 34.08%, monounsaturated FA by 40.37%, polyunsaturated FA by 17.88%, milk concentration of omega-3 by 115%, rumenic acid (CLA) by 16.50%, oleic acid by 44.87% and h/H milk index by 94.44% compared with the control treatment. Thus, the inclusion of canola oil in the diet of lactating dairy cows makes the milk fatty acid profile nutritionally healthier for the human diet; however, the lactating performance of dairy cows is reduce.

## Introduction

The fatty acid profile of the human diet has changed during the evolution of food patterns, as the diet of primitive societies was very different from the present. Due to the requirements for practicality and fast meal preparation, the intake of industrial foods, which are rich in saturated and omega-6 fatty acids, has become a necessity. As a result, the ingestion of natural foods (vegetables, fruits and fishes) that are important sources of omega-3 fatty acids is reduced. It is estimated that the omega-6/omega-3 ratio of diets in humans that lived in the period before industrialization was approximately 1:1 to 2:1, but many countries currently register a dietary omega-6/omega-3 ratio approximately 10:1 to 20:1, with an incidence of 50:1. Therefore, contrary to primitive societies, current human diets have high concentrations of saturated and omega-6 fatty acids but are deficient in omega-3, which are factors that are associated with the development with coronary heart disease and other non-infectious diseases [1, 2].

Previous studies indicated that when the dietary omega-6/omega-3 ratio was 4:1, there was a reduction in mortality by 70% in patients with cardiovascular disease, while ratios of 3 to 4:1 reduced the inflammation resulting from rheumatoid arthritis, and a ratio of 5:1 reduced the symptoms caused by asthma. When the omega-6/omega-3 ratio was 10:1, these symptoms were intensified [3, 4]. Thus, in an attempt to reduce the ingestion of saturated fat acids and the omega-6/omega-3 ratio of their diet, many people reduce the intake of ruminant-derived food products (e.g., beef and milk) because these foods generally have high saturated fatty acids and low omega-3 concentrations.

However, ruminant products, such as bovine milk, are well accepted worldwide. It is a complete food for human nutrition, has high biological value, is easily converted to various derivatives, and has many other benefits to human health. Additionally, the milk fat composition may be altered by the fatty acid composition in the diet of dairy cows. Previous studies have reported that the inclusion of vegetable oils in the diet of dairy cows may change the milk fatty acid profile, increasing unsaturated fatty acids and reducing the low-chain and saturated fatty acids [5, 6, 7].

Among the vegetable oils available for feeding dairy cows, canola oil has the highest content of unsaturated fatty acids (approximately 90%), mainly oleic (C18:1 cis-9) (51%), linoleic (C18:2 cis-9 cis-12) (25%) and alpha-linolenic acids (C18:3, omega-3) (14%). In a review performed by [8] some studies have shown that the inclusion of canola oil in the human diet contributed to reducing the incidence of cardiovascular diseases by regulating plasma lipids and lipoproteins, probably due to the high dietary concentration of oleic and omega-3 fatty acids. These fatty acids have anti-inflammatory properties, and may contribute to changes blood concentration of low-density lipoprotein (LDL), probably due a reduction in LDL synthesis, an increase in the rate of catabolism of LDL, or due both ways. It was reported that omega-3 fatty acids have the capacity to suppress the hepatic lipogenesis through reducing levels of sterol receptor element binding protein–1c, the up-regulating fatty oxidation in the liver and skeletal muscle through peroxisome proliferator–activated receptors activation, and also enhancing flux of glucose to within glycogen cells through down-regulation of hepatocyte nuclear factor–4α [9]. Otherwise, oleic fatty acid is the only long chain fatty acid that may increases the expression of genes linked to complete oxidation of fatty acids. Therefore, oleic fatty acid may promote protective effects against insulin resistance, inflammation and dyslipidemias. Moreover, with a higher rate of oxidation resulting from oleic acid it is possible that this fatty acid will also contribute to balance body weight in overnutrition states by the raised energy expenditure [8].

According to [10] the inclusion of canola in dairy cow diets reduced the milk concentrations of palmitic (C16:0) and palmitoleic (C16:1) fatty acids and increased the stearic (C18:0), oleic (C18:1), linoleic (C18:2) and gadoleic (C20:1) fatty acids in the milk fat content. Therefore, the use of canola oil in the diet of dairy cows can alter the milk fatty acid profile and render it adequate for human health in addition to meeting the basic nutritional functions of milk. However, few studies have evaluated the optimal levels of canola oil inclusion in the diet of dairy cows to maximize the milk synthesis of unsaturated fatty acids, especially omega-3 and oleic acid, without resulting in health problems for the dairy cows. Thus, the present study aimed to evaluate the effect of canola oil inclusion in the diet of dairy cows on milk production and composition and its fat quality for the human diet.

## Materials and Methods

### Experimental design and animals

The experiment was conducted at the Department of Animal Science of the Faculty of Animal Science and Food Engineering/University of São Paulo in Pirassununga, São Paulo. The study was approved by the Ethics Committee of the Department of Animal Science at the same institution, with process USP: 2013.1.475.74.7. Eighteen non-pregnant Holstein cows averaging (mean ± SD) 22 ± 4 kg/d of milk/d, 190 ± 40 days in milk (DIM) and 564 ± 70 kg of body weight (BW) were used. At the beginning of the trial, the cows were selected and balanced according to their previous milk yield, DIM and number of lactations. The experiment was conducted for three periods of 21 days, with 14 days of diet adaptation and 7 of sample collection. The cows were distributed in 6 contemporary 3x3 Latin squares consisting of 3 periods and 3 treatments: control diet (no added oil), 3% inclusion of canola oil in dry matter (DM) in the diet, and 6% inclusion of canola oil in the diet DM (Table 1). The diets were formulated according to NRC [11] recommendations.

**Table 1:** Ingredients proportion and chemical composition of the experimental diets.

Item	Inclusion of canola oil		
	Control	3%	6%
**Ingredient g/kg of DM**			
Ground corn	297.30	261.40	225.30
46%CP Soybean meal	174.70	180.60	186.70
NaCl	5.00	4.90	4.90
Mineral mixture^1^	13.80	13.80	13.80
Dicalcium phosphate	1.10	1.40	1.40
Urea	5.00	4.90	4.90
Limestone	2.30	2.40	2.40
Canola oil	-	30.00	60.00
Maize silage	500.20	500.00	500.00
**Chemical composition, g/kg of DM**			
DM	638.50	638.50	641.30
MM	39.00	38.80	38.60
OM	960.09	961.10	961.30
NDF	299.00	296.30	293.70
ADF	184.20	183.50	182.80
iADF	69.00	68.70	68.30
ADL	35.90	35.80	35.60
EE	31.90	60.10	88.20
CP	170.50	170.20	169.90
TC^2^	758.40	730.80	703.10
NFC^3^	459.30	434.40	409.40
TDN^4^	669.30	691.40	727.80
NE_l_, Mcal/Kg^5^	15.20	15.70	16.60

^1^Mineral mixture composition per kilogram: 242 g of Ca [minimum (min)], 30 mg of Co (min), 1,008 mg of Cu (min), 80 g of S (min), 390 mg of Fl (max), 39 g of P (min), 60 mg of I (min), 20 g of Mg (min), 2,998 mg of Mn (min), 1,100 mg of monensin sodium (min), 30 mg of Se (min), 4,032 mg of Zn (min), 400,000 IU of vitamin A (min), 40,000 IU of vitamin D3 (min), and 1,450 IU of vitamin E (min). MM = mineral matter; OM = organic matter; NDF = neutral detergent fiber ADF = acid detergent fiber; iADF = indigestible ADF; ADL = acid detergent lignin; EE = ether extract; CP = crude protein

^2^TC = total carbohydrates [12]

^3^NFC = no fibrous carbohydrates [13]

^4^TDN = total digestible nutrients [12]

^5^NE_l_ = net energy for lactation [11].

The animals were housed in individual stalls to evaluate the dry matter intake. The cows were fed according to the intake on the previous day to keep daily orts of 5 to 10% of the amount offered. The diet was provided once daily at 06:30 where the mixture of forage and concentrate was performed manually, and the forage:concentrate ratio used was 50:50. Following the afternoon milking, a new blend was held at the feed trough of each animal to stimulate consumption. The milking was implemented twice daily at 07:00 and 15:00 in a milking-type fishbone room and piped system following the general hygiene measures with iodine pre- and post-dipping.

### Milk Sampling and Analysis

The milk production was measured daily using the electronic flow meter of the milking machine, and the results were recorded in spreadsheets. The milk yield was measured daily, and the value was used to calculate the 3.5% fat corrected milk according to [14]. Individual milk samples representative of two daily milkings were collected from days 15 to 17 of each period, chilled, and preserved with 2-bromo- 2-nitropropane-1.3-diol (0.05%, wt/vol). In these samples, analyses were performed to determine the following components: fat, protein, lactose and total solids by infrared absorption [15]. The milk urea nitrogen content was determined by an enzymatic and colorimetric methodology [16] and somatic cell count by flow cytometry [17]. The milk solids-not-fat was obtained by calculating the difference between the fat and total solids content.

### Fatty acids profile of ingredients and milk samples

Samples were collected from the ingredients that comprise the diet for the analysis of dietary fatty acid profile (Table 2) and frozen at -20° C for further analysis. The samples were subjected to the determination of fatty acids according to the methodology described by [18] for extracting the fat followed by methylation.

**Table 2:** Fatty acid composition (% of total fatty acids) of experimental diets and canola oil.

Item	Inclusion of canola oil			
	Control	3%	6%	Canola oil
C 6:0	0.0238	0.0237	0.0236	NI
C 8:0	0.0104	0.0105	0.0106	NI
C 10:0	0.0080	0.0083	0.0085	0.009
C 12:0	0.0197	0.0199	0.0200	0.018
C 12:1	0.0655	0.0655	0.0655	NI
C 13:0	0.0205	0.0205	0.0205	NI
C 13:0 Anteiso	0.1758	0.1769	0.1779	NI
C 14:0	0.1812	0.1820	0.1828	0.079
C 15:0	0.0942	0.0943	0.0944	0.016
C 15:0 Anteiso	0.0203	0.0230	0.0230	NI
C 15:0 Isso	0.0170	0.0171	0.0172	0.003
C 15:1	0.0060	0.0052	0.0045	NI
C 16:0	18.1193	17.8229	17.5189	4.789
C 16:0 Isso	0.1320	0.1352	0.1383	NI
C 16:1 *c*-9	0.0565	0.0538	0.0512	0.218
C 17:0	0.0397	0.0393	0.0389	0.019
C 17:0 Isso	0.0660	0.0660	0.0660	NI
C 17:1	0.0205	0.0207	0.0208	0.028
C 18:0	2.6762	2.6890	2.7008	2.342
C 18:1 cis-9	22.2865	22.9765	23.6548	57.918
C 18:1 cis-11	1.7018	1.7563	1.8100	4.18
C 18:1 cis-12	0.7777	0.8001	0.8221	1.735
C 18:1 cis-13	0.5395	0.5541	0.5684	1.014
C 18:1 trans-10-11-12	0.0210	0.0210	0.0210	NI
C 18:2 cis-9 cis-12	43.1420	42.3598	41.5614	17.222
C 18:3 n-3	5.4583	5.6814	5.9014	7.491
C 18:3 n-6	0.2524	0.2599	0.2673	0.53
C 20:0	NI	0.0003	0.0006	0.01
C 20:1	0.3599	0.3921	0.4240	1.314
C 21:0	NI	0.0003	0.0005	0.009
C 22:0	0.2195	0.2259	0.2321	0.293
C 22:1	0.0035	0.0046	0.0058	0.038
C 23:0	0.0712	0.0718	0.0724	0.02
C 24:0	0.1398	0.1417	0.1436	0.196
C 24:1	NI	NI	NI	0.096
Saturated FA	21.929	21.663	21.385	7.765
Unsaturated FA	74.691	74.951	75.178	91.688
Sat/Unsat ratio	0.294	0.289	0.284	0.085
Monounsaturated FA	25.838	26.650	27.448	66.445
Polyunsaturated FA	48.853	48.301	47.730	25.243
Omega-3	5.458	5.681	5.901	7.491
Omega-6	0.252	0.260	0.267	0.530
ω6/ω3 ratio	0.005	0.005	0.005	0.071

NI: Not identified.

Milk samples were collected on the last day of each trial period in a collection tube without preservatives to determine the fatty acid profile. After collection, the samples were frozen at -20°C. The extraction of fat was performed using the method described by [19] and methylated using the method of [20].

The methylated samples were analyzed in a gas chromatograph (Model GC-Finnigan Focus; Thermo Finnigan, San Jose, CA, USA) with a flame ionization detector capillary column CP-Sil 88 (Varian), 100 m long with a 0.25 μm internal diameter and 0.20 μm film thickness. Hydrogen was used as a carrier gas with a flow of 1.8 mL/minute. The oven temperature program was an initial 70°C with a holding time of 4 minutes followed by 175° C (13° C/minute) with a holding time 27 minutes, 215°C (4°C/minute) with a holding time of 9 minutes and finally an increase by 7°C/minute to 230°C, standing for 5 minutes, for a total of 65 minutes. The vaporizer temperature was 250°C, and the detector temperature was 300°C.

A 1 μL aliquot of the esterified extract was injected into the chromatograph, and the identification of fatty acids was performed by a comparison of the retention times. The percentages of fatty acids were obtained via the software Chromquest 4.1 (Thermo Electron, Italy). The fatty acids were identified by comparing the retention times of the methyl esters of the samples with standard butter fatty acids. The fatty acids were quantified by normalization of the areas of the methyl esters. The standards used were from the Supelco TM Component FAME Mix, cat 18919 Supelco, Bellefonte, PA, USA.

The nutritional quality of the milk lipid fraction was evaluated by three indices from the composition data of fatty acids by the following equations:

atherogenicity index (*AI*) = [(*C*12:0 + (4 × *C*14:0) + *C*16:0)/(*ΣMUFA*) + *Σω*6 + *Σω*3); thrombogenicity index (*TI*) = (*C*14:0 + *C*16:0 + *C*18:0)/[(*ΣMUFA* × 0.5) + (0.5 × *Σω*6) + (3 × *Σω*3) + (*Σω*3 / *Σω*6)] according to [21]; and the ratio of hypocholesterolemic (h)/hypercholestero lemic (H) fatty acids (*h*/*H*) = (*C*18:1 *cis* − 9 + *C*18:2 *ω* − 6 + *C*18:3 *ω* − 3 + *C*20:5 *ω* − 3 + *C*22:6 *ω* − 3)/(*C*14:0 + 16:0) according to [22].

### Statistical analysis

The results were analyzed by the computer program Statistical Analysis System [23] (version 9.2, SAS Institute Inc., Cary, NC, USA) after verification of the normal errors and homogeneity of variances by Proc-Univariate. With the normal distribution of data, the statistical procedure was adopted in accordance with the main effects of the treatment with the Proc Mixed-Command of the SAS with a significance level of 5% according to the following model:

$$Y_{ijkl}=\;\mu +\;T_{i}+\;S_{j}+\;{Ck}_{\left(j\right)}+\;P_{l}+\;e_{ijkl}$$

where *Y*_*ijkl*_ = dependent variable; μ = overall mean; *T*_*i*_ = fixed effect of treatment *i* (3 df); *S*_*j*_ = fixed effect of Latin square *j* [1 to 6 (5 df)]; *Ck*_(*j*)_ = random effect of cow k within each Latin square [*k* = 1 to 18 (12 df)]; *P*_*l*_ = fixed effect of period l [1 to 3 (3 df)]; and *e*_*ijkl*_ = random error associated with each observation. The degrees of freedom are calculated according to the Satterthwaite method (DDFM = Satterth).

The treatment effect was decomposed into two orthogonal polynomial contrasts (linear and quadratic). The intercept and slope coefficients were obtained using the "estimate" option mixed procedure. Regression equations were chosen according to the Bayesian information criteria, standard error of the estimates and biological behavior of the data.

## Results

The milk yield (kg/d) decreased linearly according to the inclusion of canola oil in the diet of cows such that the cows fed with 6% canola oil in the diet produced 2.51 kg/d less milk than cows fed the control diet (Table 3). The milk fat yield and concentrations decreased in a quadratic and linear form, respectively, according to the inclusion of canola oil in dairy cow diets. The point of maximum reduction in the milk concentration of fat occurred with the inclusion of 4.5% oil in the diet. The milk fat yield decreased linearly as a result of the dietary inclusion of canola oil, decreasing 0.13 kg/d in the cows supplemented with 6% canola oil compared with cows fed the control diet.

**Table 3:** Effect of canola oil in the diet of lactating dairy cows on milk yield and composition.

Item	Inclusion of canola oil			Means	SEM	P-value**	
	Control	3%	6%			L	Q
Milk yield^1^ kg/d	23.50	22.46	20.99	22.28	0.69	0.0001	0.62
FCM^2^ kg/d	23.46	20.88	19.88	21.45	0.73	0.0001	0.105
Fat^3^ %	3.48	3.13	3.18	3.27	0.08	0.009	0.04
Fat^4^ kg/d	0.79	0.69	0.66	0.71	0.03	0.0002	0.18
CP^5^ %	3.58	3.73	3.90	3.72	0.06	0.0001	0.81
CP kg/d	0.82	0.80	0.81	0.82	0.02	0.58	0.55
Lactose %	4.44	4.46	4.45	4.45	0.03	0.83	0.51
Lactose ^6^ kg/d	1.04	1.01	0.93	1.002	0.03	0.001	0.34
SNF^7^ %	8.95	9.19	9.30	9.15	0.07	0.0003	0.43
SNF^8^ kg/d	2.09	2.06	1.96	2.04	0.05	0.005	0.405
TS %	12.34	12.32	12.60	12.41	0.13	0.16	0.34
TS^9^ kg/d	2.88	2.70	2.62	2.74	0.08	0.001	0.42
MUN, mg/dL	19.58	18.58	18.91	18.94	0.36	0.23	0.16

** L = probability of linear effect, Q = probability of quadratic effect. FCM = 3.5% fat-corrected milk; SNF = Solids not-fat; TS = Total solids; SCC = Somatic cell count; MUN = Milk urea nitrogen. IOC = Inclusion of canola oil. Equations:

^1^*Y* = 23.57 (*SE* = 1.16)− 0.42 (*SE* = 0.08) × *IOC* (%)

^2^*Y* = 23.24 (*SE* = 1.27)− 0.59 (*SE* = 0.09) × *IOC* (%)

^3^*Y* = 3.48 (*SE* = 0.17)− 0.18 (*SE* = 0.06) × *IOC* (%) + 0.02 (*SE* = 0.01) × *IOC*^2^ (%^2^)

^4^*Y* = 0.78 (*SE* = 0.05)− 0.02 (*SE* = 0.005) × *IOC* (%)

^5^*Y* = 3.58 (*SE* = 0.11)+ 0.05 (*SE* = 0.01) × *IOC* (%)

^6^*Y* = 1.05 (*SE* = 0.05)− 0,01 (*SE* = 0.003) × *IOC* (%)

^7^*Y* = 8.95 (*SE* = 0.12)+ 0.09. (*SE* = 0.05) × *IOC* (%)

^8^*Y* = 2.10 (*SE* = 0.10)− 0.02 (*SE* = 0.007) × *IOC* (%)

^9^*Y* = 2.87 (*SE* = 0.145)− 0.04 (*SE* = 0.012) × *IOC* (%).

The milk crude protein (CP) concentration increased linearly with the inclusion of canola oil in the diet, resulting in 0.32% more CP in the milk when cows were fed the 6% canola oil diet than when they were fed with control diet. However, the milk CP yield kg/d was not different between treatments. The increase in the CP concentration of milk may be due to the reduced milk production according to the inclusion of canola oil concentrating the CP.

The milk lactose concentration did not differ between treatments, but the lactose yield decreased linearly with the inclusion of canola oil, and cows fed with 6% canola oil in their diet decreased their lactose production by 0.11 kg/d compared with the cows fed with the control diet.

The total solids concentration did not differ between treatments. However, the total solids production was reduced by the inclusion of canola oil in the diet as a result of the reduction in milk fat and lactose.

The milk concentration of solids not-fat (SNF) increased linearly according to the inclusion of canola oil in the diet, while the production of SNF was reduced. This result probably occurred in response to the lower milk production in the cows fed diets with the inclusion of 6% canola oil compared with the treatment control. The milk SNF yield decreased similarly to milk production. The milk urea nitrogen (MUN) did not differ between treatments.

Milk fat is naturally composed of high concentrations of short- and medium-chain fatty acids. However, when canola oil was included in the diet of lactating cows, the milk fat composition exhibited another profile, as canola oil is composed mostly of unsaturated fatty acids. In the present study, canola oil showed a higher proportion of oleic acid (57.9%) and C18:2 cis-9 cis-12 (17.22%), a precursor of CLA C18:2 cis-9 trans-11 and alpha-linolenic acid (7.49%).

There is evidence that some fatty acids of animal products such as milk and meat from ruminants have specific physiological benefits, enrich these products and are extremely important to human health. Among these fatty acids, with the inclusion of canola oil in the diet as 6% of the DM, there was an increase of 9.86 g/100 g FA oleic acid, 0.086 g/100 g FA CLA C18:2 cis-9 trans-11 and 0.16 g/100 g of FA alpha-linolenic acid (major fatty acids that compose the series omega-3) (Tables 4 and 5).

**Table 4:** Effect of canola oil in the diet of lactating dairy cows on the saturated milk fatty acids profile (g/100g FA).

Item	Inclusion of canola oil			Mean	SEM	P-value**	
	Control	3%	6%			L	Q
C4:0^1^	2.30	1.91	1.68	1.95	0.08	<0.0001	0.436
C6:0^2^	1.60	1.17	0.96	1.23	0.06	<0.0001	0.061
C8:0^3^	1.06	0.69	0.56	0.76	0.04	<0.0001	0.008
C10:0^4^	2.42	1.56	1.24	1.72	0.08	<0.0001	0.002
C11:0^6^	0.09	0.04	0.03	0.06	0.007	<0.0001	0.068
C12:0^7^	3.45	2.35	1.96	2.57	0.10	<0.0001	0.0009
C13:0^9^	0.19	0.13	0.11	0.14	0.009	<0.0001	0.140
C13:0 Anteiso^10^	0.10	0.07	0.06	0.08	0.005	<0.0001	0.012
C13:0 Iso^11^	0.036	0.034	0.027	0.03	0.001	<0.0001	0.171
C14:0^12^	11.88	9.82	8.61	10.05	0.24	<0.0001	0.061
C14:0 Iso^13^	0.037	0.030	0.027	0.03	0.001	0.001	0.397
C15:0^15^	1.29	0.98	0.82	1.03	0.05	<0.0001	0.339
C15:0 Anteiso^16^	0.48	0.39	0.31	0.39	0.01	<0.0001	0.653
C15:0 Iso^17^	0.22	0.18	0.16	0.19	0.006	<0.0001	0.340
C16:0^18^	30.45	24.84	22.32	25.69	0.60	<0.0001	0.001
C16:0 Iso	0.21	0.20	0.19	0.19	0.01	0.256	0.936
C17:0^20^	0.44	0.34	0.28	0.36	0.01	<0.0001	0.164
C17:0 Iso^21^	0.22	0.19	0.17	0.19	0.006	<0.0001	0.415
C18:0^23^	7.97	11.22	11.77	10.31	0.37	<0.0001	0.002
C20:0^34^	0.09	0.15	0.16	0.13	0.006	<0.0001	0.0002
C21:0	0.005	0.006	0.005	0.005	0.0004	0.751	0.379
C22:0	0.01	0.02	0.02	0.02	0.001	0.129	0.550
C23:0	0.011	0.012	0.009	0.011	0.0009	0.141	0.104
C24:0^41^	0.020	0.025	0.027	0.024	0.001	0.0004	0.186

** L = probability of linear effect. Q = probability of quadratic effect. IOC = Inclusion of canola oil. Equations:

^1^*Y* = 2.27 (*SE* = 0.13)− 0.1044 (*SE* = 0.01863)× *IOC* (%)

^2^*Y* = 1.56 (*SE* = 0.08)− 0.1073 (*SE* = 0.01196)× *IOC* (%)

^3^*Y* = 1.01 (*SE* = 0.06)− 0.08340 (*SE* = 0.009465)× *IOC* (%)

^4^*Y* = 2.31 (*SE* = 0.11)− 0.1952 (*SE* = 0.01812)× *IOC* (%)

^6^*Y* = 0.08 (0.02)− 0.00897 (*SE* = 0.001890)× *IOC* (%)

^7^*Y* = 3.33 (*SE* = 0.18)− 0.2475 (*SE* = 0.02227)× *IOC* (%)

^9^*Y* = 0.19 (*SE* = 0.03)− 0.01330 (*SE* = 0.002715)× *IOC* (%)

^10^*Y* = 0.10 (*SE* = 0.009)− 0.00797 (*SE* = 0.000961)× *IOC* (%)

^11^*Y* = 0.04 (*SE* = 0.004)− 0.00152 (*SE* = 0.000330)× *IOC* (%)

^12^*Y* = 11.73 (*SE* = 0.35)− 0.5427 (*SE* = 0.04444)× *IOC* (%)

^13^*Y* = 0.04 (*SE* = 0.004)− 0.00158 (*SE* = 0.000454)× *IOC* (%)

^15^*Y* = 1.26 (*SE* = 0.12)− 0.07641 (*SE* = 0.01495)× *IOC* (%)

^16^*Y* = 0.48(*SE* = 0.03)− 0.02891 (*SE* = 0.003460)× *IOC* (%)

^17^*Y* = 0.22 (*SE* = 0.01)− 0.01013 (*SE* = 0.001994)× *IOC* (%)

^18^*Y* = 29.87 (*SE* = 0.67)− 1.3417 (*SE* = 0.09978)× *IOC* (%)

^20^*Y* = 0.44 (*SE* = 0.02)− 0.02696 (*SE* = 0.002401)× *IOC* (%)

^21^*Y* = 0.22 (*SE* = 0.01)− 0.00959 (*SE* 0.002020)× *IOC* (%)

^23^*Y* = 8.44 (*SE* = 0.72)+ 0.6303 (*SE* = 0.09155)× *IOC* (%)

^34^*Y* = 0.10 (*SE* = 0.01)+ 0.01114 (*SE* = 0.001571)× *IOC* (%)

^41^*Y* = 0.02 (*SE* = 0.002) + 0.001109 (*SE* = 0.000270) × *IOC* (%).

**Table 5:** Effect of canola oil in the diet of lactating dairy cows on the unsaturated milk fatty acids profile (g/100g FA).

Item	Inclusion of canola oil			Mean	SEM	P-value**	
	Control	3%	6%			L	Q
C10:1^5^	0.31	0.19	0.15	0.21	0.01	<0.0001	0.003
C12:1^8^	0.12	0.08	0.06	0.08	0.005	<0.0001	0.003
C14:1 cis9^14^	1.65	1.41	1.19	1.37	0.08	0.0001	0.898
C16:1 cis-9^19^	2.63	2.21	1.91	2.21	0.12	<0.0001	0.694
C17:1^22^	0.16	0.15	0.11	0.14	0.008	0.010	0.423
C18:1 cis-9^24^	21.97	28.57	31.83	27.62	0.66	<0.0001	0.0004
C18:1 cis-11^25^	1.61	2.30	2.75	2.24	0.09	<0.0001	0.270
C18:1 cis-12^26^	0.68	0.86	1.05	0.87	0.03	<0.0001	0.931
C18:1 cis-13^27^	0.49	0.56	0.67	0.57	0.01	<0.0001	0.405
C18:1 cis15^28^	0.04	0.11	0.19	0.12	0.01	<0.0001	0.550
C18:1trans-10,11,12^29^	1.75	2.80	3.23	2.64	0.16	<0.0001	0.083
C18:1 trans-16^30^	0.17	0.21	0.25	0.21	0.008	<0.0001	0.894
C18:1 trans-6,7,8,9^31^	0.52	0.91	1.28	0.92	0.06	<0.0001	0.870
C18:2 cis-9 cis-12	1.16	1.34	1.33	1.29	0.06	0.112	0.261
C18:2 cis-9 trans-11^32^	0.521	0.520	0.607	0.530	0.035	0.032	0.126
C18:2 n-6	0.035	0.033	0.040	0.035	0.003	0.345	0.345
C18:3 n-3^33^	0.19	0.36	0.35	0.30	0.01	<0.0001	0.003
C18:3 n-6	0.032	0.028	0.028	0.03	0.001	0.352	0.438
C20:1^35^	0.05	0.15	0.19	0.13	0.01	<0.0001	0.003
C20:2^36^	0.01	0.01	0.009	0.01	0.0007	0.001	0.289
C20:3 n-3	0.0018	0.0016	0.0010	0.0014	0.0002	0.224	0.688
C20:3 n-6^37^	0.06	0.05	0.04	0.05	0.002	<0.0001	0.255
C20:4^38^	0.15	0.12	0.10	0.12	0.05	<0.0001	0.123
C20:5	0.012	0.011	0.011	0.011	0.0007	0.679	0.778
C22:1^39^	0.009	0.01	0.02	0.01	0.001	<0.0001	0.848
C22:2	0.0018	0.0006	0.0009	0.0011	0.0002	0.093	0.119
C22:5^40^	0.037	0.034	0.026	0.033	0.001	<0.0001	0.035
C22:6	0.0035	0.0033	0.0025	0.0031	0.0003	0.466	0.149

** L = probability of linear effect. Q = probability of quadratic effect. IOC = Inclusion of canola oil. Equations:

^5^*Y* = 0.29 (*SE* = 0.01)− 0.02695 (*SE* = 0.003061)× *IOC* (%)

^8^*Y* = 0.12 (*SE* = 0.008)− 0.01051 (*SE* = 0.001088)× *IOC* (%)

^9^*Y* = 0.19 (*SE* = 0.03)− 0.01330 (*SE* = 0.002715)× *IOC* (%)

^10^*Y* = 0.10 (*SE* = 0.009) - 0.00797 (*SE* = 0.000961)× *IOC* (%)

^11^*Y* = 0.04 (*SE* = 0.004)− 0.00152 (*SE* = 0.000330)× *IOC* (%)

^12^*Y* = 11.73 (*SE* = 0.35)− 0.5427 (*SE* = 0.04444)× *IOC* (%)

^13^*Y* = 0.04 (*SE* = 0.004)− 0.00158 (*SE* = 0.000454)× *IOC* (%)

^14^*Y* = 1.63 (*SE* = 0.14)− 0.07407 (*SE* = 0.01660)× *IOC* (%)

^9^*Y* = 2.60 (*SE* = 0.20)− 0.1185 (*SE* = 0.02642)× *IOC* (%)

^22^*Y* = 0.16 (*SE* = 0.01)− 0.00692 (*SE* = 0.002857)× *IOC* (%)

^24^*Y* = 22.59 (*SE* = 0.85)+ 1.6312 (*SE* = 0.1001)× *IOC* (%)

^25^*Y* = 1.66 (*SE* = 0.18)+ 0.1886 (*SE* = 0.02049)× *IOC* (%)

^26^*Y* = 0.68 (*SE* = 0.04) + 0.06143 (*SE* = 0.008259)× *IOC* (%)

^27^*Y* = 0.48 (*SE* = 0.05) + 0.03046 (*SE* = 0.005550)× *IOC* (%)

^28^*Y* = 0.04 (*SE* = 0.02)+ 0.02508 (*SE* = 0.002957)× *IOC* (%)

^29^*Y* = 1.87 (*SE* = 0.33) + 0.2451 (*SE* = 0.03506)× *IOC* (%)

^30^*Y* = 0.17 (*SE* = 0.01)+ 0.01330 (*SE* = 0.002651)× *IOC* (%)

^31^*Y* = 0.53 (*SE* = 0.09)+ 0.1265 (*SE* = 0.01586)× *IOC* (%)

^32^*Y* = 0.49 (*SE* = 0.06)+ 0.01709 (*SE* = 0.005881)× *IOC* (%)

^33^*Y* = 0.22 (*SE* = 0.03)+ 0.02756 (*SE* = 0.006386)× *IOC* (%)

^35^*Y* = 0.06 (*SE* = 0.01) + 0.02473 (*SE* = 0.001837)× *IOC* (%)

^36^*Y* = 0.01 (*SE* = 0.001) − 0.00083 (*SE* = 0.000230)× *IOC* (%)

^37^*Y* = 0.06 (*SE* = 0.003)−0.00404 (*SE* = 0.000682)× *IOC* (%)

^38^*Y* = 0.15 (*SE* = 0.01)− 0.00821 (*SE* = 0.001117)× *IOC* (%)

^39^*Y* = 0.009 (*SE* = 0.002)+ 0.002414 (*SE* = 0.000445)× *IOC* (%)

^40^*Y* = 0.03 (*SE* = 0.003) − 0.00177 (*SE* = 0.000260) × *IOC* (%).

The inclusion of 6% of canola oil in the diet of lactating cows linearly reduced the milk concentration of short-chain FA by 41.42%, medium-chain FA by 27.32%, saturated FA by 20.24%, saturated/unsaturated ratio by 39.20%, omega-6/omega-3 ratio by 39.45%, milk atherogenicity index by 48.36% and thrombogenicity index by 39.86% compared with the cows fed with the control diet (Table 6).

**Table 6:** Effect of canola oil inclusion in the diet of lactating dairy cows on the nutritional quality of lipid fraction of milk (g/100g FA).

Item	Inclusion of canola oil			Mean	SEM	P-value**	
	Control	3%	6%			L	Q
Short Chain FA^1^	11.25	7.93	6.59	8.54	0.36	<0.0001	0.004
Medium Chain FA^2^	49.66	40.69	36.09	41.36	0.95	<0.0001	0.0019
Long Chain FA^3^	38.44	50.63	56.09	49.57	1.29	<0.0001	0.003
Saturated FA^4^	64.84	55.97	51.71	56.93	1.04	<0.0001	0.0221
Unsaturated FA^5^	35.68	43.91	47.84	43.12	1.13	<0.0001	0.0388
Sat/Unsat ratio ^6^	1.76	1.26	1.07	1.33	0.01	<0.0001	0.0723
Monounsaturated FA^7^	32.32	41.40	45.37	39.24	1.08	<0.0001	0.0035
Polyunsaturated FA^8^	2.18	2.54	2.57	2.50	0.07	0.0165	0.1666
Rumenic acid^9^	0.521	0.520	0.607	0.530	0.035	0.032	0.126
Oleic acid^10^	21.97	28.57	31.83	27.62	0.66	<0.0001	0.0004
Omega-3^11^	0.20	0.38	0.43	0.33	0.02	<0.0001	0.0017
Omega-6^12^	1.80	2.33	2.39	2.18	0.07	<0.0001	0.0360
ω6/ω3 ratio ^13^	9.86	6.43	5.97	7.46	0.44	<0.0001	0.0399
Atherogenicity^14^	2.44	1.55	1.26	1.72	0.08	<0.0001	0.0004
Thrombogenicity ^15^	2.91	2.05	1.75	2.20	0.08	<0.0001	0.0022
h/H^16^	0.54	0.83	1.05	0.82	0.03	<0.0001	0.0747

** L = probability of linear effect. Q = probability of quadratic effect. Short Chain FA = C4:0-C12:0; Medium Chain FA = C12:1-C17:1; Long Chain FA = C18:0-C24:0;

Omega-3 = (C18:3n3+C20:3n3+C20:5+C22:6); Omega-6 = (C18:2cis9cis12+ C18:2cis9 trans11+C18:2+C18:3n6+C20:2+C20:3n6+C22:2); Omega-6/omega-3 ratio; h/H = hypocholesterolemic and hypercholesterolemic. IOC = Inclusion of canola oil.

Equations:^1^*Y* = 10.90 (*SE* = 0.47)− 0.7778 (*SE* = 0.07139)× *IOC* (%)

^2^*Y* = 48.66 (*SE* = 0.9664)− 2.2023 (*SE* = 0.1437)× *IOC* (%)

^3^*Y* = 40.24 (*SE* = 1.49) + 2.8067 (*SE* = 0.2342)× *IOC* (%)

^4^*Y* = 63.80 (*SE* = 1.1512)− 2.1377 (*SE* = 0.1872) × *IOC* (%)

^5^*Y* = 37.16 (*SE* = 1.2895) + 1.8996 (*SE* = 0.2037)× *IOC* (%)

^6^*Y* = 1.71 (*SE* = 0.09646)− 0.1107 (*SE* = 0.01708)× *IOC* (%)

^7^*Y* = 32.96 (*SE* = 1.1629) + 2.2449 (*SE* = 0.1830)× *IOC* (%)

^8^*Y* = 2.29 (*SE* = 0.1864) + 0.05343 (*SE* = 0.02317)× *IOC* (%)

^9^*Y* = 0.49 (*SE* = 0.06) + 0.01709 (*SE* = 0.005881)× *IOC* (%)

^10^*Y* = 22.59 (*SE* = 0.85) + 1.6312 (*SE* = 0.1001)× *IOC* (%)

^11^*Y* = 0.22 (*SE* = 0.02477) + 0.03810 (*SE* = 0.004697)× *IOC* (%)

^12^*Y* = 1.87 (*SE* = 0.2165) + 0.09855 (*SE* = 0.02226)× *IOC* (%)

^13^*Y* = 9.32 (*SE* = 0.9235)− 0.6664 (*SE* = 0.1418)× *IOC* (%)

^14^*Y* = 2.33 (*SE* = 0.09627)− 0.1942 (*SE* = 0.01783)× *IOC* (%)

^15^*Y* = 2.79 (*SE* = 0.1062)− 0.1883 (*SE* = 0.01919)× *IOC* (%)

^16^*Y* = 0.55 (*SE* = 0.03891) + 0.08419 (*SE* = 0.003637)× *IOC* (%).

The 6% treatment linearly increased the milk concentration of long-chain FA by 45.91%, unsaturated FA by 34.08%, monounsaturated FA by 40.37%, polyunsaturated FA by 17.88%, the concentration of omega-3 in milk by 115%, rumenic acid (CLA) by 16.50%, oleic acid by 44.87% and the h/H ratio by 94.44% compared with cows in the control group. The h/H ratio increased from 0.54 g/100 g of FA in the control group to 1.05 g/100 g of FA in milk from the 6% treatment.

## Discussion

The inclusion of canola oil in the diet of dairy cows reduced the milk concentrations of saturated fatty acids, the omega-6/omega-3 ratio and the milk indices of atherogenicity and thrombogenicity. As well as, increased the milk content of unsaturated fatty acids, omega-3, CLA, and oleic acid and the h/H ratio, thereby improving the nutritional milk quality as these fatty acids play essential roles in human health.

The supplementation of canola oil as 3% and 6% of dietary DM linearly reduced the milk yield probably due to the high lipid content in the diet (6.01% and 8.82% EE of DM, respectively) compared with the control treatment (3.19% EE of DM). Additionally, this oil presents a high content of unsaturated fatty acids (91.7%). Unsaturated lipids are toxic to rumen microorganisms, which may reduce the intake and digestibility of dry matter and nutrients modifying the ruminal fermentation. This reduction of intake and ruminal fermentation can reduce the rate of digestion and consequently the flow of nutrients to the mammary gland, which in turn reduces milk production.

The milk fat was reduced when canola oil was included in the diet. The supplementation of unsaturated lipids in the diet of dairy cows may reduces milk fat content due to the production of trans fatty acids by incomplete ruminal biohydrogenation of dietary unsaturated fatty acids. When the trans fatty acids are absorbed into the gut and reach the mammary gland, the expression of lipogenic enzymes (e. g., acetyl-CoA carboxylase and fatty acid synthase) that act in “de novo” synthesis of fatty acids can be reduced. The “de novo” synthesis is responsible for the formation of fatty acids of up to 16 carbons, reducing the milk fat concentrations of short- and medium-chain fatty acids, which provide approximately 60% of the total milk fat acid content [24]. In our study, we observed a linear reduction in fatty acid concentration up to C:17 and a linear increase in fatty acids above C:18 with the canola oil inclusion, indicating a lower lipogenic enzyme activity in “de novo” synthesis (Tables 4 and 5).

According to [24], among the trans fatty acids that act in milk fat depression, the C18:2 trans10 cis12, C18:1 trans10 and C:18 trans10:1 trans11 fatty acids formed by partial biohydrogenation of fatty acids are present in the rumen. Although the milk concentration of C18:2 trans10 cis12, which is mainly responsible for the fat reduction in milk, was not detected in this study, we observed a linear increase in the fatty acids C18:1 trans10 and C18:1 trans11, which can also be associated with the reduction in milk fat. In the present study, Table 4 shows that the fatty acids C18:1 trans10, 11 and 12 were not separately identified. However, there is a linear increase in these fatty acids with the inclusion of canola oil, configuring an increase of 84.57% in the 6% treatment compared with the control treatment. These results corroborate the literature for the milk fat reduction.

Canola oil contains a large amount of unsaturated fatty acids, and the oil used in this study contained 91.7% of unsaturated fatty acids. Many of these fatty acids must pass through the rumen without suffering biohydrogenation or the partial biohydrogenation of fatty acids, resulting in formation of intermediate FA of ruminal biohydrogenation as trans FA, e.g., C18:2 trans10 cis12 and C18:1 trans11.

The partial biohydrogenation of fatty acids are confirmed by observing the fatty acid profile present in milk (Tables 4 and 5). The stearic acid (C18:0), the final step for complete biohydrogenation of linoleic acid (C18:2), increased by 3.25 g/100 g FA in milk from the cows in the 3% treatment when compared with the control. However, in the 6% treatment there was an increase of 3.8 g/100 g FA compared with the control. This result suggests that the partial or incomplete biohydrogenation was higher because stearic acid increased only by 0.55 g/100 g FA, resulting in larger quantities of unsaturated fatty acids that followed the gastrointestinal tract flow.

These unsaturated fatty acids are absorbed through the intestinal epithelium. In the enterocytes the fatty acids are re-esterified to triacylglycerols and arranged mainly in chylomicrons. Through the lymphatic system they reach the bloodstream where, being directed to peripheral tissues such as the mammary gland, they increase the unsaturated fatty acids in milk fat [25].

Among the beneficial fatty acids to human health is the increase in oleic acid in the milk fatty acid profile (Table 6). Over the past few years, some studies have demonstrated the beneficial effects of oleic acid on human health because it has modulator effects and large physiological functions. Some suggest a beneficial effect on cancer, autoimmune and inflammatory diseases as well as the capacity of oleic acid to facilitate wound healing [26]. Oleic acid can lower blood pressure through the adrenergic activity that helps regulate blood pressure [27] as well as aid in weight loss. Additionally, according to [8], oleic acid increases the expression of genes involved in fat burning.

In a study performed by [28], people who consumed larger amounts of oleic acid had an 89% less chance of developing ulcerative colitis than those consuming lower amounts of oleic acid. Oleic acid also has a beneficial effect on type II diabetes by reversing the adverse effects of inflammatory cytokines observed in obesity and non-insulin-dependent diabetes [29]. Oleic acid, being less susceptible to oxidation damage than omega-3 fatty acids and omega-6, also replaces other omega fatty acids in cell membranes, protecting the cell membranes from free radicals and other oxidative stressors [30].

Conjugated linoleic acid (CLA) also increased with the inclusion of canola oil. CLA (C18:2 cis-9 trans-11) is a natural component of food products derived from ruminants. Studies evaluating the consumption of dairy products rich in CLA such as cheese have shown an inverse association with breast cancer and colorectal cancer in women. Animal models suggest that the mechanisms of the anti-carcinogenic effects of CLA include the modulation of eicosanoid production interference in the signaling pathway inhibition of DNA synthesis, promotion of apoptosis and angiogenesis modulation [31]. A review by [32] shows that preclinical data indicate a beneficial effect of CLA on bone health.

New evidence states that the trans fat that is naturally present in the milk and meat of ruminants is beneficial for cardiovascular health. The results of animal studies suggest that CLA also has anti-atherosclerotic properties, improves the blood lipid profile by reducing total cholesterol, triglycerides, and LDL [31].

The omega-6/omega-3 ratio in the human diet comes after many years of attempting to recapture the balance found before the industrial era when the consumption of natural food was the only or most abundant option. The search for a healthier diet has been the aim of several studies on human health in view of the numerous cases of non-communicable diseases that trigger the death of thousands of people worldwide. The milk from cows fed canola oil becomes a useful tool in this relentless pursuit of a healthier diet focused on preventing certain diseases. The lowest omega-6/omega-3 ratio, indicating a healthier food, was 5.9:1 in the 6% treatment compared with the 9.8:1 ratio of the control treatment. According to [33] the World Health Organization (WHO), together with the United Nations Food and Agriculture Organization (FAO), recommends a ratio of (5:1) up to (10:1), which are the suggested values for a balanced diet between omega-6/omega-3.

The need for balancing the omega-6/omega-3 ratio is because omega-6 presents pro-inflammatory effects, enhancing the production of cytokines with vasoconstriction, which promotes platelet aggregation. Platelet aggregation is related to the occurrence of cardiovascular, autoimmune and inflammatory diseases such as arthritis, asthma, psoriasis, lupus and ulcerative colitis. On the other hand, omega-3 fatty acids play an essential role in preventing cardiovascular diseases because it produces eicosanoids with lower inflammatory power that control the narrowing of the arterial lumen by deposition of fat in the blood vessel wall. Moreover, it has anti-inflammatory properties, promotes vasodilation and inhibits platelet aggregation, which are functions that are also related to the prevention of hypertension, atherosclerosis, hypercholesterolemia, arthritis and other autoimmune and inflammatory diseases as well as various cancers [34].

The atherogenicity and thrombogenicity indices decreased with the addition of canola oil in the diet of cows. The atherogenicity (ability to induce atherosclerosis formation) and thrombogenicity (ability to promote heart attacks and strokes) indices indicate that the smaller the values of these indices, the greater the amount of atherogenic fatty acids present in a given fat or oil, and the greater the potential for prevention against the development of coronary heart disease. There is no recommended value for these indices in dairy products; however, it is considered that the lower the value of these rates, the more favorable the AG profile to human health [35].

A study by [36] on butter made with cow's milk supplemented with 0; 1.5; 3.0 and 4.5% sunflower oil showed a linear reduction (P <0.0001) in the atherogenicity (AI) and thrombogenicity (TI) indices with the inclusion of sunflower oil: AI: 4.02; 2.70; 1.80; 1.41 and TI: 4.68; 3.43; 2.50 and 2.05, respectively. Oil inclusion results in rate reductions, thus improving the butter quality and making it healthier. These results corroborate the data from this study, where the inclusion of canola oil linearly reduced these rates. However, due to the higher level and the difference in canola versus sunflower oil composition, the cows supplemented with 6% canola oil in their diet showed the best results with an AI of 1.26 and a TI of 1.75.

One study [7] using flaxseed (1.2 kg/day) and encapsulated fish oil (200 g/day) for lactating cows to assess the profile of fatty acids in milk as well as the atherogenicity and thrombogenicity indices. The AI in the control treatment, fish oil treatment, and flaxseed treatment was 2.119, 2.093 and 1.859, respectively. The TI for these treatments was as follows: 2.50, 2.41 and 2.31, respectively. In the study by [7], flaxseed had the lowest results within the evaluated treatments, but these indices were numerically lower in our study.

The h/H ratio increased with the oil inclusion. This index considers the functional activity of fatty acids in the metabolism of lipoproteins that transport cholesterol in plasma, where the type and quantity are related to a higher or lower risk of cardiovascular disease incidence [22]. The ratio of h/H is contrary to the atherogenicity and thrombogenicity indices because the higher the h/H, the better the nutritional value of the oil or fat and the lower the risk of cardiovascular disease incidence. The canola oil content linearly increased the h/H index by 0.54 g/100 g of AG in the control to 1.05 g/100 g fatty acids in the milk from cows fed with the 6% inclusion of canola oil in their diets. These results corroborate those of [36], who reported a linear increase in the h/H index in the butter with sunflower oil included in the diet of cows. The treatments were 0, 1.5, 3 and 4.5%, and the results were as follows: 0.30, 0.45, 0.62 and 0.70, respectively.

Foods of animal origin, such as milk, have often been cast as villains due to the amount of saturated fatty acids, which have always been identified as precursors of cardiovascular disease. However, animal products are no longer excluded from the diet because the fatty acid profiles of the foods derived from ruminants can be modified over time through changes in the animal diet, with canola oil as an excellent alternative for beneficial results.

## Conclusions

The inclusion of canola oil in the diet of lactating dairy cows linearly reduces the milk yield and daily fat output, the milk fat concentration of saturated fatty acids, the milk omega-6/omega-3 ratio and the atherogenicity and thrombogenicity indices. Moreover, the milk concentration of omega-3, CLA, and oleic acid and the h/H ratio of milk are improved with the inclusion of canola oil in the diets of lactating dairy cows, which makes the fatty acid profile of the milk nutritionally healthier for human diets.
